# Area-level deprivation and adverse childhood experiences among high school students in Maryland

**DOI:** 10.1186/s12889-022-13205-w

**Published:** 2022-04-23

**Authors:** Shaheen Kurani, Lindsey Webb, Kechna Cadet, Ming Ma, Marianne Gibson, Nikardi Jallah, Ju Nyeong Park, Renee M. Johnson

**Affiliations:** 1 Division of Health Care Delivery Research, Mayo Clinic, Rochester, MN USA; 2 Department of Mental Health, Johns Hopkins Bloomberg School of Public Health, Baltimore, USA; 3 Department of Community & Behavioral Health, Colorado School of Public Health, Denver, CO USA; 4 Maryland Department of Health, Opioid Operational Command Center, Crownsville, MD USA; 5 Maryland Department of Health, Prevention & Health Promotion, Baltimore, MD USA; 6 Department of Health, Behavior and Society, Johns Hopkins Bloomberg School of Public Health, Baltimore, USA

**Keywords:** Adverse childhood experiences, Social determinants of health, Area-level deprivation, Rurality

## Abstract

**Background:**

Nearly one-half of Americans have been exposed to at least one adverse childhood experience (ACE) before turning 18, contributing to a broad array of problems spanning physical health, mental and behavioral health, and psychosocial functioning.

**Methods:**

This was a cross-sectional, survey research study, using 2018 data from a state adolescent health surveillance system, i.e., Maryland Youth Risk Behavior Survey/Youth Tobacco Survey. The population-based sample of Maryland high school students (*n* = 41,091) is representative at the state and county levels. The outcome variables included five binary measures of ACEs (i.e., food insecurity, parental substance use/gambling, parental mental illness, family member in jail/prison, and caregiver verbal abuse), and number of ACEs. The main exposure variable, area-level socioeconomic disadvantage, was assessed at the county level using a continuous measure of the area deprivation index (ADI). Additional covariates included: rural county status, age, race/ethnicity, sex, and sexual or gender minority (SGM) status. We used mixed-effect multivariate logistic regression to estimate the odds of ACEs in association with socioeconomic deprivation. Models were adjusted for all covariates.

**Results:**

County-level ADI was associated with 3 of the 5 ACES [i.e., food insecurity (OR = 1.10, 95% CI: 1.07–1.13), parental substance use/gambling (OR = 1.05, 95% CI: 1.02–1.07), and incarceration of a family member (OR = 1.14, 95% CI: 1.09–1.19)]; and with having at least one ACE (i.e., OR = 1.08, 95% CI: 1.05–1.10). Odds of reporting at least one ACE were higher among girls, older adolescents (i.e., aged 16 and ≥ 17 relative to those aged ≤ 14 years), and among SGM, Black, and Latinx students (all ORs > 1.20).

**Conclusions:**

ACEs greatly increase risk for adolescent risk behaviors. We observed an increased likelihood of adversity among youth in more deprived counties and among Black, Latinx, or SGM youth, suggesting that social and structural factors play a role in determining the adversity that youth face. Therefore, efforts to address structural factors (e.g., food access, family financial support, imprisonment as a sanction for criminal behavior) could be a critical strategy for primary prevention of ACEs and promoting adolescent health.

**Supplementary Information:**

The online version contains supplementary material available at 10.1186/s12889-022-13205-w.

## Introduction

Although adversity has long been a focus of research on the etiology of behavioral problems, much of the scientific thinking on the link between adversity and health comes from the 1998 “Adverse Childhood Experiences Study,” which investigated health in association with childhood exposure to substance use, mental illness, violence, criminal behavior, and child maltreatment (including psychological, physical, and sexual abuse) [1, 2]. The study demonstrated that there are strong associations between adverse childhood experiences (ACEs) and a broad array of problems spanning physical health, mental and behavioral health, and psychosocial functioning [3–8]. The link between ACEs and health problems has been attributed to prolonged activation of the stress-response system, leading to maladaptive coping, impulsivity, and impairments in learning, attention, and decision-making [9, 10]. Current research indicates that nearly one-half of Americans have at least one ACE before turning 18 years old, and that the annual costs to society exceeds a billion dollars [8, 11].

It has now been two decades since the ACE Study, and a strong body of work demonstrates that adversity is a critical factor in adolescent risk behaviors, including violence, school failure, and substance use [11–15]. Broader recognition of how adversity shapes adolescent behavioral health has led to an increased focus on preventing risk behaviors by attending to underlying trauma [8, 16–18], typically through interventions and services at the community and organizational levels. Interventions include strategies such as connecting youth to supportive adults through mentoring programs, implementing mindfulness training in schools, and screening for ACEs by pediatricians and other health care providers [2, 17, 18]. Although important, these types of psychosocial interventions do not address societal factors that increase risk for youth adversity [19]. Initiatives that target structural factors have the potential to prevent children from experiencing ACEs. For example, policies to reduce deportations, replace incarceration with alternate criminal sanctions, and broaden the economic safety-net could decrease the number of children who face adverse experiences such as parental separation and food insecurity [1, 20, 21]. The combination of structural interventions to prevent youth adversity and psychosocial approaches to attend to youth who have experienced adversity could be powerfully effective at ensuring the health and well-being of adolescents. Therefore, an important next step for preventing ACEs – and the focus of this study – is to identify whether societal factors increase risk for ACEs.

Living in an area characterized by socioeconomic disadvantage increases risk for a range of health and social problems [22, 23] and may also increase risk for youth adversity. Research shows that individuals living in areas of greater deprivation are more likely to experience morbidity and mortality, even after adjusting for individual-level sociodemographic factors [23, 24]. Several studies indicate that low socioeconomic position is associated with increased risk for ACEs [25–29]. People from low-income households are at greater risk for experiencing specific types of ACEs [26] and for overall greater numbers of ACEs [29]; low socioeconomic position is also a positive moderator of the association between ACEs and health outcomes [28]. However, studies investigating whether area-level socioeconomic disadvantage is associated with increased risk for ACEs are surprisingly sparse. It is not known whether the deprivation-adversity link would hold if disadvantage were conceptualized as a feature of the social environment, versus an individual or family characteristic. Understanding the relationship between area-level disadvantage and adversity would provide clues about how structural factors influence risk for ACEs.

The purpose of this study is to investigate the association between county-level disadvantage and ACEs among a representative, population-based sample of Maryland high school students. We explored five ACEs: food insecurity, parental substance use/gambling, parental mental illness, family member in jail/prison, and caregiver verbal abuse. This set of ACEs is common and strongly associated with later problems in life, including mental disorders [30, 31]. Given that risk for negative outcomes is higher among those who reported more ACEs, we also examined how many of the ACEs students reported [30]. We used the area deprivation index (ADI) to measure county-level disadvantage; ADI is a validated, composite indicator of socioeconomic disadvantage that spans four domains: income, housing, employment, and education [32]. Our findings will provide needed information about area-level disadvantage and youth adversity, and may provide a foundation for research to contextualize the drivers of disparities in ACEs.

## Methods

### Sample

We conducted a secondary analysis of 2018 surveillance data on Maryland adolescents using the Maryland Youth Risk Behavior Surveillance System and the Youth Tobacco Survey (MD-YRBS/YTS) [33]. MD-YRBS/YTS was conducted with coordination from the CDC, and the data collection instrument is based on standard national surveys [34, 35].

A two-stage cluster sample design was used to produce a sample of Maryland high school students (9^th^-12^th^ graders) that was representative of students at the county and state levels. Schools were randomly selected with probability proportional to enrollment size (stage 1), and then classrooms were randomly sampled within schools (stage 2). Data were weighted to represent the population and to adjust for non-response. The overall response rate (i.e., the product of response rates at the school and student levels) was > 60% for each county (*n* = 41,091).

### Outcome Variables

The survey included five binary questions that assessed adversity, including: food insecurity (*“During the past 12 months, how often did the food your family bought not last and they did not have money to get more?”*), parental substance use/gambling (*“Have you ever lived with anyone who was an alcoholic or problem drinker, used illegal street drugs, took prescription drugs to get high, or was a problem gambler?”*), parental mental illness (*“Have you ever lived with anyone who was depressed, mentally ill, or suicidal?*), family member in jail/prison (*“Has anyone in your household ever gone to jail or prison?”*), and caregiver verbal abuse (*“Does a parent or other adult in your home regularly swear at you, insult you, or put you down?”*). These items were adapted from the Behavioral Risk Factor Surveillance System (BRFSS) ACE module [36, 37], and are conceptually similar to items from the ACE Study. We created two additional measures on number of ACEs; the first indicated whether respondents reported 0, 1, 2, or 3 or more ACEs, and the second was a binary measure indicating 1 or more ACEs versus none.

### Predictor Variables

ADI, a composite measure of area-level socioeconomic disadvantage [38–40], was calculated for all 24 Maryland jurisdictions (i.e., 23 counties and Baltimore City, which functions as a county) [39]. The ADI is constructed using 17 variables from 5-year American Community Survey (ACS) estimates [41–43]. Kurani et al. (2021) provide detailed information on the methodology for ADI derivation and the factor analysis approach used to assign weights to each variable (eTable 1). The ADI score was continuous, with higher scores indicating greater deprivation and scaled by 10 in the model.

Covariates included rurality and demographic factors. County designations as rural or not were based on classifications assigned by the Rural Maryland Council [44]. Demographic variables included age ($\le$ 14, 15, 16, $\ge$ 17), sex (male/female), race/ethnicity (non-Hispanic White; non-Hispanic Black; Hispanic/Latinx, regardless of race; and all other groups), and sexual or gender minority (SGM) status (yes/no). The ‘all other’ category included non-Hispanic students who were Asian, American Indian/Alaska Native, Native Hawaiian/Pacific Islander, or Multiracial. Students who reported they were gay/lesbian, bisexual, or ‘unsure’ as their sexual orientation and/or who identified as transgender were classified as SGM.

We restricted the analytical sample to those with complete data on study variables. Using the complete sample as a denominator, less than 8.5% of students had missing data on any specific ACE, i.e., 6.6% for food insecurity (*n* = 2,727), 8% for parental substance use/ gambling (*n* = 3,274), 8.1% for parental mental illness (*n* = 3,338), 7.5% for family member in jail/ prison (*n* = 3,085), and 8.4% for caregiver verbal abuse (*n* = 3,461). Because of missing data, we used separate samples for analyses of each of the five ACEs and for number of ACEs.

To characterize ACEs among the sample, we estimated the prevalence and 95% confidence interval for each ACE and for number of ACEs (i.e., none, 1, 2, 3 or more) for the total sample. We also present prevalence estimates by race and ethnicity, age, sex, and SGM status. To assess associations between ADI and ACEs, we conducted mixed-effect multivariable logistic regression models. This included six models, one for each specific ACE and a sixth predicting at least one ACE (versus none). Models were adjusted for county rural status, age, race/ethnicity, sex, and SGM status. Analyses were conducted with the survey analysis procedures in SAS v9.4 and Stata 15.1, which facilitated use of sample weights and accounted for complex sampling structure. We used the Huber-White robust standard errors clustered at the county level to account for nesting within counties.

## Results

For all six samples (i.e., each of the 5 ACEs and a sixth sample measuring 1 or more ACEs versus none), approximately 45% of the students were White, 30% were ≥ 17 years of age, 50% were girls, and 18% were SGM (Table 1). The most commonly reported ACE among White and Latinx students was parental mental illness, whereas having a family member in jail/prison was the most commonly reported ACE among Black students (Table 2). With each increase in age category, respondents were more likely to report having experienced any of the five ACEs. Girls had a higher prevalence than boys of four of the five ACEs; boys were more likely than girls to report having a family member in jail/prison. SGM students had a higher prevalence of all five ACEs relative to cisgender, heterosexual students. The most commonly reported ACE reported among SGM students was parental mental illness.

**Table 1:** Description of samples, Maryland high school students, 2018 (*n* = 41,091)

	**Food Insecurity**		**Parental Substance Use/Gambling**		**Parental Mental Illness**		**Family Member in Jail/Prison**		**Caregiver Verbal Abuse**		**All ACEs**	
	**(*n* = 35,347)**		**(*n* = 34,921)**		**(*n* = 34,877)**		**(*n* = 35,034)**		**(*n* = 34,735)**		**(*n* = 33,828)**	
	**%**	*n*	**%**	*n*	**%**	*n*	**%**	*n*	**%**	*n*	**%**	*n*
**Race/Ethnicity**												
White	44.6%	20,577	44.8%	20,419	44.9%	20,394	44.9%	20,459	45.0%	20,318	45.6%	19,966
Black	33.4%	6,885	33.1%	6,738	33.0%	6,726	33.2%	6,794	33.0%	6,693	32.4%	6,380
All Other	12.9%	4,772	13.0%	4,725	13.0%	4,721	12.9%	4,732	13.0%	4,697	13.1%	4,582
Latinx	9.1%	3,113	9.0%	3,039	9.1%	3,036	9.0%	3,049	9.0%	3,027	8.9%	2,900
**Age, years**												
≤ 14	20.1%	7,745	20.1%	7,642	20.1%	7,623	20.0%	7,674	20.0%	7,596	20.0%	7,393
15	25.3%	9,682	25.3%	9,576	25.3%	9,535	25.3%	9,600	25.3%	9,489	25.3%	9,266
16	24.7%	8,937	24.7%	8,834	24.7%	8,849	24.8%	8,879	24.8%	8,811	24.9%	8,592
≥ 17	29.9%	8,983	30.0%	8,869	29.9%	8,870	29.8%	8,881	30.0%	8,839	29.7%	8,577
**Sex**												
Girls	49.6%	17,084	49.3%	16,799	49.5%	16,791	49.5%	16,897	49.5%	16,749	49.2%	16,177
Boys	50.4%	18,263	50.7%	18,122	50.5%	18,086	50.5%	18,137	50.5%	17,986	50.8%	17,651
**Sexual or Gender Minority**												
No	82.4%	29,615	82.6%	29,304	82.7%	29,273	82.6%	29,400	82.7%	29,182	82.9%	28,484
Yes	17.6%	5,732	17.4%	5,617	17.3%	5,604	17.4%	5,634	17.3%	5,553	17.1%	5,344

**Table 2:** Prevalence estimate (and 95% confidence interval) of individual ACEs, by race/ethnicity, sex, age category, and sexual and gender minority (SGM) status

	**Food Insecurity**		**Parental Substance Use/Gambling**		**Parental Mental Illness**		**Family Member in Jail/Prison**		**Caregiver Verbal Abuse**	
	**(*n* = 5,486)**		**(*n* = 9,083)**		**(*n* = 11,093)**		**(*n* = 8,612)**		**(*n* = 7,688)**	
	**%**	**95% CI**	**%**	**95% CI**	**%**	**95% CI**	**%**	**95% CI**	**%**	**95% CI**
**Total**	16.5	[15.4,17.6]	23.7	[22.8,24.7]	29.7	[28.8,30.6]	23.3	[22.3,24.4]	20.7	[19.8,21.5]
**Race**										
White	10.0	[9.2,10.9]	24.1	[23.2,25.1]	33.7	[32.6,34.9]	17.1	[16.2,18.0]	19.1	[18.1,20.2]
Black	24.9	[22.8,27.0]	22.9	[21.4,24.6]	24.0	[22.6,25.6]	33.2	[31.1,35.4]	21.5	[19.7,23.5]
All Other	13.0	[11.2,15.0]	20.7	[18.1,23.6]	27.6	[25.4,29.9]	17.7	[15.2,20.6]	20.9	[19.0,23.0]
Latinx	22.3	[19.0,26.0]	29.2	[26.0,32.6]	33.4	[29.7,37.3]	26.1	[23.2,29.3]	24.6	[22.4,27.0]
**Age, years**										
≤ 14	13.8	[12.2,15.4]	22.5	[20.8,24.3]	26.7	[25.0,28.5]	22.3	[20.3,24.4]	21.2	[19.2,23.4]
15	15.7	[14.2,17.2]	21.9	[20.3,23.5]	27.8	[26.5,29.2]	24	[22.1,26.1]	21.3	[19.5,23.2]
16	16.6	[14.9,18.4]	24.4	[22.9,26.0]	31.5	[29.8,33.2]	24.3	[22.7,26.1]	21.1	[19.3,22.9]
≥ 17	18.8	[16.9,20.9]	25.6	[23.4,28.0]	31.8	[30.3,33.4]	22.6	[20.5,24.9]	19.4	[18.2,20.7]
**Sex**										
Boys	16.1	[14.7,17.6]	22.5	[21.3,23.9]	24.9	[23.5,26.3]	23.5	[22.0,25.2]	18.2	[16.9,19.4]
Girls	16.8	[15.8,18.0]	24.9	[23.7,26.2]	34.4	[33.3,35.5]	23.1	[21.9,24.4]	23.1	[22.1,24.2]
**SGM Status**										
No	15.0	[13.9,16.1]	22	[21.1,23.0]	26.3	[25.4,27.3]	22.1	[21.0,23.3]	18.2	[17.3,19.1]
Yes	23.5	[21.5,25.6]	32	[30.1,33.9]	45.7	[43.4,48.0]	29.0	[27.1,31.0]	32.6	[30.8,34.5]

The *p* value was < 0.001 for all tests of statistical significance except: race/ethnicity and caregiver verbal abuse (*p* = 0.002); age with gambling/ substance use (*p* = 0.017), jail/prison (*p* = 0.386), and caregiver verbal abuse (*p* = 0.329); sex with food insecurity (*p* = 0.323), gambling/substance use (*p* = 0.005), and jail/prison (*p* = 0.686)

One-fourth of the students reported just one ACE, whereas 15% reported two and 15.6% reported three or more (Table 3). Black and Latinx students had the lowest prevalence of reporting zero ACEs, ~ 37% for both groups. Boys were more likely than girls to report zero ACEs (47.9% vs. 39.7%), and heterosexual, cisgender students were more likely than their SGM peers to report zero ACEs (47.9% vs 27.1%). Twenty-six percent of SGM students reported 3 or more ACEs, an estimate higher than all other demographic groups.

**Table 3:** Prevalence estimate (and 95% confidence interval) for number of ACEs, by race/ethnicity, sex, age, and sexual and gender minority (SGM) status, *n* = 33,828

	**None**		**1 ACE**		**2 ACEs**		** ≥ 3 ACEs**	
	**(*n* = 14,528)**		**(n = 8,237)**		**(n = 5,095)**		**(*n* = 5,968)**	
	**%**	**95% CI**	**%**	**95% CI**	**%**	**95% CI**	**%**	**95% CI**
**Total**	43.7	[42.5,45.0]	25.6%	[24.8,26.4]	15.0%	[14.3,15.7]	15.6%	[14.8,16.5]
**Race**								
White	47.5	[46.2,48.8]	24.9	[23.9,25.9]	13.0	[12.4,13.6]	14.7	[13.9,15.5]
Black	37.4	[35.4,39.6]	28.3	[26.8,30.0]	17.9	[16.4,19.5]	16.3	[14.6,18.2]
All Other	50.4	[46.6,54.1]	23.0	[21.1,24.9]	12.9	[11.3,14.6]	13.8	[11.9,15.9]
Latinx	37.9	[34.6,41.2]	23.4	[20.6,26.5]	17.9	[15.8,20.1]	20.9	[18.1,24.0]
**Age, years**								
≤ 14	47.0	[44.4,49.6]	25.1	[23.6,26.6]	13.3	[12.1,14.7]	14.6	[13.0,16.2]
15	44.7	[42.9,46.4]	25.6	[24.2,27.2]	15.0	[13.7,16.3]	14.7	[13.5,16.1]
16	42.4	[40.2,44.5]	25.5	[23.8,27.3]	15.9	[14.5,17.3]	16.3	[14.8,17.8]
≥ 17	42.0	[39.5,44.5]	26.1	[24.8,27.4]	15.4	[14.0,16.9]	16.6	[15.0,18.3]
**Sex**								
Boys	47.9	[46.0,49.7]	24.5	[23.3,25.7]	14.0	[13.0,15.0]	13.7	[12.5,14.9]
Girls	39.7	[38.3,41.2]	26.7	[25.6,27.8]	16.0	[15.1,16.9]	17.6	[16.6,18.6]
**SGM Status**								
No	47.2	[45.9,48.4]	25.2	[24.3,26.0]	14.2	[13.5,14.9]	13.5	[12.7,14.3]
Yes	27.1	[24.8,29.6]	27.9	[26.0,29.9]	18.8	[17.3,20.4]	26.1	[24.2,28.2]

The *p* values for associations between number of ACEs and race/ethnicity, sex, and SGM status were all < 0.001, the *p* value for the association between number of ACEs and age was 0.036

Multiple regression analyses show that, after adjustment for demographic factors, students in more deprived counties were at an increased likelihood of reporting at least one ACE (Table 4). County-level ADI was associated with increased odds of food insecurity (OR = 1.10, 95% CI: 1.07–1.13), parental substance use/gambling (OR = 1.05, 95% CI: 1.02–1.07), and having a family member who had been to prison/jail (OR = 1.14, 95% CI: 1.09–1.19). Rural county status was associated with increased odds of reporting parental substance use/gambling (OR = 1.24, 95% CI: 1.05–1.47), having a family member in jail/prison (OR = 1.35, 95% CI: 1.04–1.76), and caregiver verbal abuse (OR = 1.25, 95% CI: 1.08–1.46).

**Table 4:** Association between ACEs and county- and individual-level factors, Odds Ratios (and 95% Confidence Intervals)

	**Food Insecurity**			**Parental Substance Use/ Gambling**			**Parental Mental Illness**			**Family Member in Jail/Prison**			**Caregiver Verbal Abuse**			**Any ACES** **(1 + vs 0)**		
	**OR**	**LL**	**UL**	**OR**	**LL**	**UL**	**OR**	**LL**	**UL**	**OR**	**LL**	**UL**	**OR**	**LL**	**UL**	**OR**	**LL**	**UL**
**LEVEL 2**																		
** Area-Deprivation Index (ADI) Score**	**1.10**	**1.07**	**1.13**	**1.05**	**1.02**	**1.07**	1.02	1.00	1.04	**1.14**	**1.09**	**1.19**	1.03	1.00	1.05	**1.08**	**1.05**	**1.10**
**Rural County**																		
No (Ref.)																		
Yes	1.16	0.97	1.38	**1.24**	**1.05**	**1.47**	1.08	0.99	1.19	**1.35**	**1.04**	**1.76**	**1.25**	**1.08**	**1.46**	1.08	0.94	1.23
**LEVEL 1**																		
**Race/Ethnicity**																		
White (Ref)																		
Black	**2.83**	**2.36**	**3.39**	0.94	0.86	1.02	**0.61**	**0.54**	**0.68**	**2.29**	**2.04**	**2.58**	**1.16**	**1.06**	**1.26**	**1.39**	**1.28**	**1.50**
All Other	**1.55**	**1.36**	**1.76**	0.94	0.83	1.06	**0.79**	**0.69**	**0.91**	**1.35**	**1.09**	**1.68**	**1.24**	**1.16**	**1.34**	1.00	0.86	1.16
Latinx	**2.75**	**2.21**	**3.42**	**1.40**	**1.25**	**1.58**	1.01	0.87	1.17	**2.03**	**1.80**	**2.29**	**1.43**	**1.33**	**1.55**	**1.57**	**1.44**	**1.70**
**Age**																		
≤ 14 (Ref)																		
15	**1.16**	**1.04**	**1.30**	0.97	0.82	1.15	1.07	0.97	1.18	**1.12**	**1.02**	**1.23**	1.01	0.94	1.08	1.11	0.98	1.25
16	**1.24**	**1.05**	**1.48**	**1.15**	**1.02**	**1.29**	**1.33**	**1.23**	**1.44**	**1.13**	**1.01**	**1.26**	1.01	0.88	1.16	**1.24**	**1.16**	**1.32**
≥ 17	**1.43**	**1.27**	**1.62**	**1.20**	**1.09**	**1.31**	**1.32**	**1.20**	**1.47**	0.99	0.89	1.10	0.89	0.72	1.11	**1.23**	**1.18**	**1.29**
**Sex**																		
Boys (Ref.)																		
Girls	0.95	0.87	1.04	1.06	0.97	1.16	**1.44**	**1.30**	**1.59**	**0.89**	**0.80**	**0.99**	**1.21**	**1.06**	**1.37**	**1.25**	**1.17**	**1.34**
**SGM**																		
No (ref.)																		
Yes	**1.76**	**1.59**	**1.95**	**1.65**	**1.44**	**1.90**	**2.20**	**1.93**	**2.51**	**1.50**	**1.29**	**1.74**	**2.09**	**1.81**	**2.41**	**2.31**	**2.16**	**2.47**

ADI. *OR* Adjusted odds ratio, *UL* Upper limit, and *LL *Lower limit

Associations between ACEs and race/ethnicity held even after adjustment for county-level ADI and rural status. Compared to White students, Black students were 183% more likely to experience food insecurity, 129% more likely to have a family member in jail/prison, and 16% more likely to experience caregiver verbal abuse. By contrast, Black students were 40% less likely to report parental mental illness than their White peers. Compared to White students, Latinx students had greater odds of reporting food insecurity (OR = 2.75, 95% CI: 2.21–3.42), parental substance use/gambling (OR = 1.40, 95% CI: 1.25–1.58), family member in jail/prison (OR = 2.03, 95% CI: 1.80–2.29), and caregiver verbal abuse (OR = 1.43, 95% CI: 1.33–1.55).

The odds of reporting all five ACEs were particularly high among SGM students relative to heterosexual, cisgender students, and there were also noteworthy associations between ACEs and age, sex, and rural county status. SGM students were 131% more likely to report at least one ACE and were at least 50% more likely to report each of the five ACEs. Living in a rural county was associated with increased risk for reporting parental substance use/gambling (OR = 1.24, 95% CI: 1.05–1.47), family member incarceration (OR = 1.35, 95% CI: 1.04–1.76), and caregiver verbal abuse (OR = 1.25, 95% CI: 1.08–1.46), Compared to those aged 14 or younger, students aged 15, 16, or 17 or older had significantly higher odds of reporting four of the five ACEs (the exception being caregiver verbal abuse). Compared to boys, girls were less likely to report having a family member in jail/prison (OR = 0.89, 95% CI: 0.80–0.99), but more likely to report parental mental illness (OR = 1.44, 95% CI: 1.30–1.59), caregiver verbal abuse (OR = 1.21, 95% CI: 1.06–1.37), and at least one ACE (OR = 1.25, 95% CI: 1.17–1.34).

## Discussion

To examine how social factors shape risk for adversity, we investigated county-level socioeconomic disadvantage in association with ACEs among a statewide sample of Maryland high school students. We found that ADI – an indicator of county-level socioeconomic deprivation – was associated with significantly higher odds of reporting three ACEs (i.e., food insecurity, parental substance use/gambling, and having a family member in jail/prison), but was not associated with the other two ACEs (i.e., parental mental illness and caregiver verbal abuse). ADI was also associated with significantly higher odds of reporting at least one ACE. As described in more detail below, the prevalence of specific types of ACEs was higher among youth in rural counties, Black and Hispanic/Latinx youth, and SGM youth. Increased likelihood of ACEs among youth in more disadvantaged counties, in rural counties, and in marginalized populations suggests that social and structural factors shape risk for experiencing adversity.

Rural county status was associated with increased risk for parental substance use/gambling, family member in jail/prison, and caregiver verbal abuse. The fact that it was not associated with increased odds for reporting at least one ACE suggests rural status is linked to specific types of adversity, rather than to adversity in general. Increased risk for these three ACEs could relate to the fact that rural communities have been especially hard hit by substance use problems, especially opioids [45–49]), as well as by child abuse and neglect, and mass incarceration [50, 51]. Compounding the problem, rural parents and families face isolation and have limited access to social support, family services, and treatment for substance use problems [52–54].

Black and Latinx students were significantly more likely than White students to report having experienced at least one ACE, highlighting the racialized nature of exposure to adversity among Maryland adolescents. Compared to their White peers, Black and Latinx students were more likely to report food insecurity, that a family member had been to jail/prison, and caregiver verbal abuse. Latinx students were 40% more likely than White students to report parental substance use/gambling; although there was no difference among Black versus White students. Disparities in ACEs likely stem from race-based inequities in social institutions, including education, criminal justice, and workplaces [55, 56]. Notably, Black youth were less likely to report parental mental illness compared to White youth, which could reflect cultural differences in what constitutes mental illness and lower help-seeking behaviors, or an actual difference in prevalence [57].

SGM status was the only demographic factor that was strongly and significantly associated with each of the 5 ACEs and with number of ACEs. These findings are consistent with previous literature in adult [58] and adolescent [59, 60] samples, and there are several explanations for this phenomenon. Given the enduring cultural stigma surrounding their identity, many SGM adolescents may experience rejection and abuse from their parents [61, 62]. SGM adolescents may be kicked out of their home or run away because of abuse [63]. Those youth who attempt to preserve the parental relationship by not disclosing may experience anxiety, isolation, and limited support in navigating peer relationships. Parental rejection of SGM youth may contribute to their increased likelihood for caregiver verbal abuse, parental mental illness, and food insecurity, but it is not entirely clear why SGM adolescents report higher levels of family member incarceration or parental substance use/gambling than their heterosexual, cisgender peers.

Setting the county as the contextual unit of analysis is a strength of our study. Maryland is different from many US states in that counties represent a meaningful unit of the lived experience and the municipal jurisdiction for public school systems, in addition to being a geographic unit for data on population statistics. The MD YRBS/YTS is unique among adolescent health surveillance systems in that it is powered to be representative at the county level, and it includes items on ACEs. Thus, we leveraged a unique opportunity to investigate the association between county-level socioeconomic disadvantage and adversity in a population-based sample of adolescents. Given that data are from a single year in one state, additional studies are required to draw more definitive conclusions about socioeconomic disadvantage and adversity in adolescence. It will also be important to determine how the nature of our observations might change with more detailed measurement of ACEs, such as asking about additional ACEs or clarifying chronicity of experiences.

ACEs can have negative, long-lasting effects on development and can lead to maladaptive coping mechanisms, including substance use [5–7, 9, 10, 64]. Given the substantial public health burden of ACEs, primary prevention is a key national priority. We demonstrate that area-level socioeconomic disadvantage is linked to the adversity that youth experience, and we also show that there are disparities in adversity among Black, Latinx, rural, and SGM youth. Our findings suggest that strategies for the primary prevention of ACEs should address structural factors such as food access, family financial support, imprisonment as a sanction for criminal behavior, strategies to change cultural attitudes toward LGBT youth and to support their families, and access to family services and behavioral health care.

Importantly, identifying specific structural targets that will move the needle on youth adversity requires careful consideration of the mechanisms through which macro-level phenomena effect families and youth. States and other municipal agencies should prioritize comprehensive assessment of ACEs and how to prevent them. In Maryland, Governor Hogan recently signed an Executive Order proclaiming that it is the State’s policy to promote the understanding of the impacts of adversity, toxic stress, and trauma on development, and to promote resilience through protective factors and programs. Efforts such as this will enable locales to develop trauma-informed programming, interventions, surveillance and screening, and have great potential for reducing the disparate burden of ACEs.

## Supplementary Information

**Additional file 1.** 

**Additional file 2.** 

## Data Availability

Availability of data and materials Maryland YRBS/YTS data were used for this study. The authors do not have permission to make them publicly available, but they are available from the Maryland Department of Health upon reasonable request. Data requests should be sent by email to mdh.yrbs_ytsdatarequest1@maryland.gov. Requests must include a description of what data is being requested and what the data will be used for. The county-level dataset used in the current study is available from the corresponding author on reasonable request.
