# Supplementary material for: Area-level deprivation and adverse childhood experiences among high school students in Maryland
Source: BMC Public Health. 2022 Apr 23;22:811. doi: 10.1186/s12889-022-13205-w (PMC9034595; doi:10.1186/s12889-022-13205-w)
Supplement: Supplementary file 1 — Additional file 1. [file 12889_2022_13205_MOESM1_ESM.docx]

**Area-Level Deprivation and Adverse Childhood Experiences among High School Students in Maryland**

***Background***

The Maryland Department of Health (MDH) and the Maryland State Department of Education (MSDE) work together to conduct one health-related student survey in the fall of even calendar years. The survey is a combination of the Youth Risk Behavior Survey (YRBS) and Youth Tobacco Survey (YTS). The core of the survey is the YRBS, supplemented by questions from the YTS and other surveys. An independent professional survey contractor administers the anonymous and confidential surveys, collects answer sheets, and sends them to the CDC. The CDC scans the answer sheets, creates the database, and conducts primary data analysis using the CDC’s YRBS protocols.

***Consent and Survey Procedures***

Prior to survey administration, parents are sent an “opt-out” form with a cover letter that includes a brief description of the survey, a list of FAQs, and a link to the full instrument. The letter also includes information about: risks and benefits, privacy, options for participation, and contact information to survey personnel. To opt their child out of participation, parents must sign and return the “[opt-out](https://health.maryland.gov/phpa/ohpetup/SiteAssets/Pages/YTRBS/2021%20MD%20YRBS-YTS%20Permission%20Form.pdf)” form. Briefly, parents are provided with the following information:

- The survey was designed by the CDC to identify risk behaviors that may include safety behaviors such as use of helmets and seat belts, depression, and mental health, use of tobacco, alcohol, or other drugs, nutrition and physical activity, and sexual behavior. The survey takes no more than one class period (45 minutes) to complete.
- The survey has been designed to protect your child’s privacy. The survey is anonymous, and students will not put their names on the survey. Also, no school or student will ever be mentioned by name in a report of the results.
- Doing this survey will cause little or no risk to your child. The only potential risk is that some students might find certain questions to be sensitive. If your child is not comfortable answering a question, he or she may skip it. In addition, students may stop participating in the survey at any point without penalty.
- Although your child may not benefit immediately from taking part in the survey, all children will ultimately benefit from the information collected that will guide the development and implementation of State and local programs designed to increase their health.
- However, the survey is voluntary. No action will be taken against the school, you, or your child, if your child does not take part.

When students are given the survey in school, trained facilitators describe the survey and indicate that participation is voluntary. Students are provided with the following instructions:

- This survey is about health behavior. It has been developed so you can tell us what you do that may affect your health. The information you give will be used to improve health education for young people like yourself.
- DO NOT write your name on this survey. The answers you give will be kept private. No one will know what you write.
- Completing the survey is voluntary. Whether or not you answer the questions will not affect your grade in this class. If you are not comfortable answering a question, just leave it blank.
- The questions that ask about your background will be used only to describe the types of students completing this survey. The information will not be used to find out your name. No names will ever be reported.

***Declarations***

- Ethics approval and consent to participate
  - *All methods for the YRBS were performed in accordance with relevant guidelines and regulations. The YRBS/YTS was reviewed and approved by the Maryland Department of Health (MDH) Institutional Review Board (IRB), the agency responsible for reviewing and approving all proposed research projects involving human subjects throughout MDH (FWA0002813​). Informed consent was collected from all survey participants and their parents/guardians.*
  - *The Institutional Review Board at Johns Hopkins Bloomberg School of Public Health designated this study as exempt from review.*
- Consent for publication *Not applicable*
- Availability of data and materials
  - *Data from the Maryland YRBS/YTS are available by email request from Maryland Department of Health. Requests must include a description of what the data will be used for, and users must agree to data use and data security requirements.*
- Competing interests
  - *The authors declare that there are no conflicts of interest or competing interests.*
- Funding
  - The YRBS/YTS is sponsored by the Maryland Department of Health (MDH) in collaboration with the Maryland State Department of Education (MSDE).
  - The Division of Adolescent and School Health (DASH), within the Centers for Disease Control and Prevention (CDC), provides funding to the Maryland State Department of Education to establish and strengthen systematic procedures to collect and report Youth Risk Behavior Survey (YRBS).
  - This study was funded by a SPARK grant from the Bloomberg American Health Initiative (PI: Johnson).
  - Dr. Lindsey Webb’s work was supported by the Drug Dependence Epidemiology Training Grant (National Institute on Drug Abuse [NIDA], T32DA007292-25, MPI: Johnson & Maher).
  - Centers for Disease Prevention and Control (R49CE003090, U48DP006384).
  - The content is solely the responsibility of the authors and does not necessarily represent the official views of the National Institutes of Health or the Centers for Disease Control and Prevention.
- Authors' contributions (This statement must exactly match on Editorial submission system and in the manuscript)
  - RMJ developed the initial idea, JNP and RMJ secured funding for the research, and NJ provided guidance about the dataset. SK and RMJ took a lead role in drafting the manuscript, with KC and LW assisting. NJ provided detailed information about survey methods, adn NJ and MG summarized implications of the research for Maryland youth. SK took a lead role in data analysis, with assistance from MM and LW. All authors provided feedback on the analytical strategy and drafts. All authors have read and approved the final manuscript.
- Acknowledgements *We thank all study respondents and study staff for their time and for their contribution to science.*
