# Supplementary material for: Area-level deprivation and adverse childhood experiences among high school students in Maryland
Source: BMC Public Health. 2022 Apr 23;22:811. doi: 10.1186/s12889-022-13205-w (PMC9034595; doi:10.1186/s12889-022-13205-w)
Supplement: Supplementary file 2 — Additional file 2. [file 12889_2022_13205_MOESM2_ESM.docx]

**Supplemental Material**

**SPECIFICATION OF ADI FOR MARYLAND COUNTIES**

The Area-Deprivation Index (ADI) is derived based on 17 indicators of socioeconomic status from the U.S. Census; the variables represent material deprivation, poverty, joblessness, housing expenses, etc (see eTable 1). ADI is a standardized score with a mean of 100; 99% of US counties’ scores fall between 40 and 160. The range of ADI scores for Maryland counties was 34.9-118.4. Our specification of ADI was different than other studies, which usually include national and regional samples comprised of hundreds of counties. ADI is typically divided into nationally-standardized quintiles (with 100 as the mean) and treated as an ordered variable. Because Maryland has 24 counties, splitting them into standardized quintiles would result in some of the quintiles representing just one county, and others representing the majority of counties. Specifically, the number of counties within quintiles would be: 1 in Quintile 5 (Somerset), 1 in Quintile 4 (Baltimore City), 3 in Quintile 3 (Allegany, Dorchester, Caroline), 4 in Quintile 2 (Kent, Wicomico, Garrett, Washington), and 15 in Quintile 1 (all remaining counties). We used the continuous ADI score and scaled it by 10 for ease of interpretation. This approach was the most appropriate for a state-level analysis that had a small number of counties, most of which were low levels of deprivation relative to the entire U.S.

**eTable 1. County-level American Community Survey (ACS) 5-year estimates and factor score coefficients**

| US Census Indicator | 2013-2017 ACS Table Reference, 5-year estimates | Factor Score Coefficient 2017 |
| --- | --- | --- |
| Median family income | B19013 | -0.16993 |
| Income disparity | B19001 | 0.06799 |
| Families below poverty level | B17010 | 0.12298 |
| % population below 150% poverty threshold | C17002 | 0.23659 |
| Single parent household with dependents <18 | B23008 | 0.04165 |
| Households without a motor vehicle | B25044 | 0.05646 |
| Households without a telephone | B25043 | 0.00892 |
| Occupied housing units without complete plumbing | B25016 | 0.02963 |
| Owner occupied housing units | B25003 | -0.00733 |
| Households with >1 person per room | B25014 | 0.03747 |
| Median monthly mortgage | B25088 | -0.13004 |
| Median gross rent | B25064 | -0.06295 |
| Median home value | B25077 | -0.0749 |
| Employed persons ≥16 in white collar occupation | C24010 | -0.01947 |
| Civilian labor force unemployed (aged ≥16) | B23025 | 0.02451 |
| Population aged ≥25 with <9yr education | B15003 | 0.01132 |
| Population aged ≥25 with at least a high school education | B15003 | -0.21015 |
